# Surgical Management of Brain Metastases in Patients Aged 80 and Above: Observations From a Limited Case Series

**DOI:** 10.7759/cureus.89602

**Published:** 2025-08-08

**Authors:** Robert Ziechmann, Samuel L Ricci, Scott Shepard

**Affiliations:** 1 Neurosurgery, Thomas Jefferson University Hospital, Philadelphia, USA; 2 Neurosurgery, Temple University Hospital, Philadelphia, USA

**Keywords:** brain metastases, craniotomy for tumor, decision-making, geriatric neurosurgery, neuro-oncology, surgical decision-making

## Abstract

Introduction

Potentially surgical brain metastases are increasingly common in patients aged 80 and older, yet the risk-benefit profile of surgical resection in this population remains inadequately defined. Surgical intervention in octogenarians carries a high risk due to systemic issues associated with advanced age and prevalent comorbidities, and data on perioperative morbidity and functional outcomes are limited.

Methods

A retrospective case series including six patients aged 80 years and older who underwent craniotomy for the resection of brain metastases at a single tertiary care center was conducted. Preoperative and postoperative functional status were assessed. Surgical complications, discharge disposition, and survival outcomes were reviewed through detailed chart analysis and follow-up data. Due to the limited sample size (N=6), no formal statistical analysis was performed.

Results

Preoperative Karnofsky Performance Status (KPS) averaged 68, and postoperative KPS was 80. One (17%) patient experienced a postoperative hemorrhage necessitating reoperation. Discharge dispositions included two (33%) patients discharged home, three (50%) patients discharged to acute rehabilitation, and one (16%) patient discharged to a skilled nursing facility; all patients discharged to outside facilities ultimately returned home. The median survival time was 13 (range: 2-48) months.

Conclusion

Surgical resection in patients over 80 years undergoing craniotomy for brain metastases is associated with elevated systemic risk related to comorbidities and systemic disease burden. However, specific patients may benefit, particularly those with large lesions with well-controlled systemic disease and limited medical comorbidity. Deaths observed in this cohort were attributable to systemic disease unrelated to the surgical intervention or intracranial disease.

Given the very small sample size, these findings are exploratory and require confirmation with larger studies. Surgical intervention for this population should be considered on a case-by-case basis, with a focus on patients who are neurologically symptomatic with good systemic disease control.

## Introduction

Metastases represent the majority of intracranial malignancies, and incidence has been increasing due to improved survival of cancer patients and screening practices [1-3]. The standard of care treatment for brain metastases involves multimodal treatment, including radiation therapy, systemic therapy, and surgery in select patients. The primary indication for surgery is for large lesions, particularly if they are symptomatic or are associated with significant edema [4].

Advanced age has been well-studied as a poor prognostic indicator in patients with other neurosurgical pathology, especially in the population 80 years or older, which has sometimes been described as "very elderly" [5]. Literature on the surgical management of brain metastases has studied patients 65 and older ("elderly" ), with few series specifically discussing very elderly patients and none of them addressing outcomes as we describe for the very elderly with brain metastases [6-10]. One study found that mortality and complications were relatively high in very elderly patients with brain metastases, glioma, meningioma, or pituitary adenoma, but did not separately report outcomes for very elderly patients with brain metastases [6]. Another found that age over 75 was not necessarily associated with worse short-term outcomes after craniotomy for the group of both gliomas and brain metastases [7].

There are no guidelines specifically addressing craniotomy for very elderly patients with surgically appropriate metastatic brain disease. Numerous studies have evaluated the role of radiation therapy, including both whole brain radiation therapy and stereotactic radiation, in the very elderly population. The data consistently suggest that radiation therapy is generally well tolerated. The role of surgery remains underexplored [10,11].

Craniotomy for tumor resection in very elderly patients has been investigated across tumor types. A retrospective cohort study demonstrated a 2.5-fold increase in mortality in octogenarians undergoing meningioma resection compared to age- and sex-matched controls, but long-term survival beyond two years was comparable [9]. Another study found comparable outcomes in very elderly patients versus patients under 65 undergoing craniotomy for benign and malignant brain tumors, but did not separately analyze patients over 80 years with intracranial metastases [10]. These findings suggest that selected very elderly patients can safely undergo craniotomy for diverse brain tumors when functional status and comorbidities are appropriately considered.

The aim of this study is to describe the oncologic outcomes, complications, and survival in a small case series of patients aged 80 and older who underwent surgical resection for brain metastases. No series to date has focused on these outcomes in this specific group.

## Materials and methods

Data collection

This retrospective case series was done under an institutional review board (IRB) for clinical outcomes in neuro-oncology. Included were patients aged 80 years and older who underwent craniotomy for the resection of brain metastases at Temple University Hospital. Decision-making for surgery included preoperative discussion at our multidisciplinary tumor board with neurosurgery, radiology, radiation oncology, and medical oncology participating. Tumor board discussion reviewed each patient's computed tomography (CT) of the head, thin-cut magnetic resonance imaging (MRI) of the brain with and without contrast, and recent (generally within the past three months) systemic imaging consisting of at least CT of the chest and CT of the abdomen and pelvis with contrast. Imaging in all cases, brain and systemic, was compared to prior imaging to evaluate for progression. Surgery was recommended for patients with solitary metastases that were large and/or symptomatic. All patients underwent formal medical risk stratification. Excluded were patients under 80 years of age, patients with multiple intracranial metastases, patients with progressive systemic disease despite treatment, and patients whose medical risk was deemed relatively high for this age group. Data collected included demographic information, including age and primary tumor type, preoperative and postoperative functional status (Karnofsky Performance Status (KPS)), surgical complications, discharge disposition (e.g., home, acute rehabilitation, and skilled nursing facility), and overall survival, including the cause of death. KPS was measured at the first clinic follow-up visit (when a standard neurological examination was also performed), typically at 2-3 weeks post-operation. Assessment of local control per our practice involved a postoperative MRI with and without contrast, generally performed at three months. Due to the limited sample size (N=6), no formal statistical analysis was performed.

Surgical management

Preoperative planning included detailed imaging review, including thin-cut CT and MRI with and without contrast, to consider tumor size, location, and proximity to eloquent cortex. Craniotomy was performed under general anesthesia with total intravenous anesthesia and intraoperative neuromonitoring (motor evoked potentials and somatosensory evoked potentials for all cases). Patients were pinned in a C clamp head holder, and neuronavigation was registered with intraoperative CT after pinning.

After verifying registration using anatomic landmarks, the borders of the tumor (as well as any cortical vessels and/or eloquent cortex) were mapped at the level of the skin, and a craniotomy just beyond the borders of the tumor was planned. When possible, linear or curvilinear incisions were planned to optimize wound healing (as opposed to question mark incisions). Craniotomy is made, navigation is again used to confirm adequate craniotomy and location of the tumor and vessels and/or eloquent cortex. If near eloquent areas and if appropriate, cortical mapping was done either using cortical stimulation or phase reversal to identify particularly primary motor and primary sensory cortex. If near the language cortex, surgical decision-making was guided by anatomy. Epidural tack-ups were generally placed. The dura is generally opened in a cruciate fashion. Corticotomy was made only wide enough to expose the whole tumor. Our approach was to perform en bloc resection by dissecting circumferentially as able with gentle use of the bipolar and microscissors. Hemostasis was generally performed with bipolar cautery and oxidized regenerated cellulose hemostatic agent. The craniotomy was replated with a plating set. After resection, intraoperative CT was again performed to evaluate the extent of resection and screen for any immediate postoperative complications.

Postoperatively, patients were monitored in the neurosurgical intensive care unit. They received steroid (dexamethasone) dosed to the extent of edema and generally tapered over the course of two weeks, which was then managed by radiation oncology as all patients underwent radiosurgery to the tumor resection bed.

## Results

**Figure 1:**
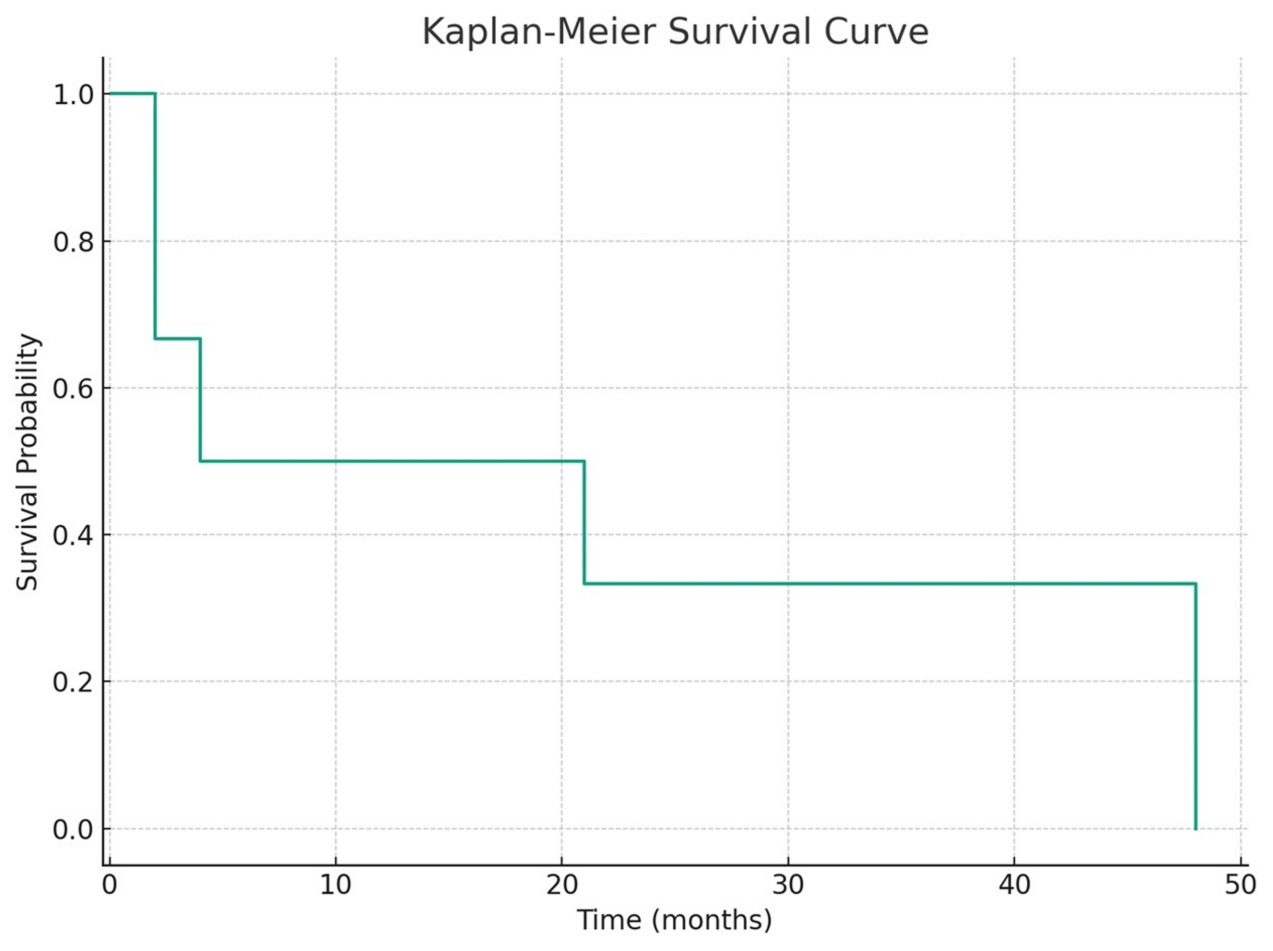
Kaplan-Meier survival curve depicting the probability of survival for very elderly patients undergoing craniotomy for brain metastasis.

Six patients with an average age of 82.8 (range: 80-85) years underwent craniotomy for a tumor, summarized in Table 1. The preoperative Karnofsky Performance Status (KPS) averaged 68 (range: 60-80), and postoperative KPS was 80 (range: 70-90). Complications included one (17%) patient with a postoperative hemorrhage requiring return to the operating room. At discharge from the admission for surgery, two (33%) patients were discharged home, three (50%) patients were discharged to acute rehabilitation, and one (16%) patient was discharged to a skilled nursing facility; all patients discharged to outside facilities ultimately were able to return home. The median survival time was 13 (range: 2-48) months. All patients (100%) had local control at first and last radiographic follow-up; no patient required further surgery or repeat radiosurgery for their metastasis treated with surgical resection. In all cases, death was associated with systemic disease, and in no cases was death attributable to complications from surgery (such as deep venous thrombosis after surgery leading to pulmonary embolism). In all cases of early mortality, death was associated with systemic infection (pneumonia or COVID-19 infection), not related to the surgery. The Kaplan-Meier survival curve for our case series is represented in Figure 1.

**Table 1:** Demographic information and clinical outcomes in patients 80 years and over undergoing craniotomy for the resection of brain metastases. NSCLC: non-small cell lung cancer, SNF: skilled nursing facility, COVID-19: coronavirus disease 2019, SCLC: small cell lung cancer, IVC: inferior vena cava

Case	Age (years)	Primary tumor	Metastasis location	Postoperative complications	Initial discharge disposition	Cause of death	Survival
1	85	NSCLC	Right parietal lobe	None	Home care	Pneumonia	21 months
2	83	NSCLC	Left temporoparietal cortex	Postoperative hematoma requiring reoperation; mild aphasia	Acute rehab	Pneumonia	2 months
3	85	Clear cell	Left cerebellar hemisphere	None	Home	Heart failure	48 months
4	80	Squamous cell lung carcinoma	Right frontal lobe	None	SNF	COVID-19 infection	2 months
5	80	Breast adenocarcinoma	Right frontal parenchyma	None	Acute rehab	Respiratory failure	48 months
6	84	SCLC	Left parieto-occipital region	Deep vein thrombosis requiring an IVC filter	SNF	Pneumonia	4 months

**Figure 2:**
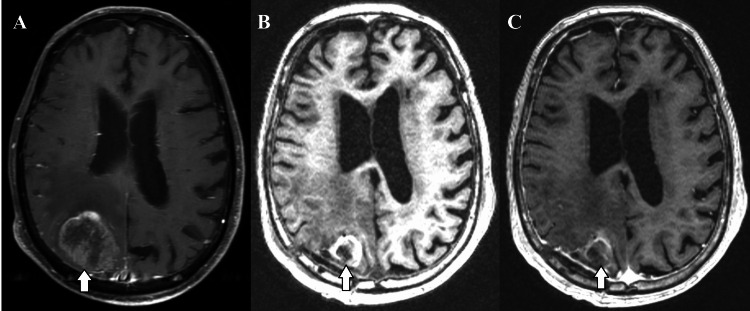
Preoperative T1-weighted MRI demonstrating the brain metastasis (A, arrow). T1-weighted MRI demonstrating resection without (B) and with (C) contrast (arrows). MRI: magnetic resonance imaging

In case 1, an 85-year-old man with stage III non-small cell lung carcinoma previously in remission presented after a fall. Brain MRI revealed a 4 cm right parietal lesion with extensive vasogenic edema (Figure 2A). Preoperative risk stratification estimated low cardiac risk. Given lesion size and symptoms, a right parietal craniotomy was performed, achieving gross total resection (Figure 2B, 2C). Pathology confirmed metastatic non-small cell lung carcinoma. Postoperatively, the patient received Gamma Knife stereotactic radiosurgery (18 Gy) to the resection cavity and was started on osimertinib for systemic therapy. At four months, the patient had no focal neurological deficit and was independent in activities of daily living. Follow-up MRI at one year showed no recurrence. The patient survived 21 months following surgery without any neurological events, eventually expiring due to pneumonia.

**Figure 3:**
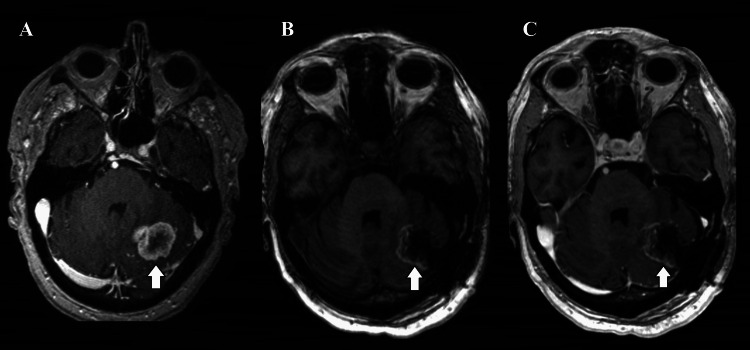
Preoperative T1-weighted MRI demonstrating the brain metastasis (A, arrow). T1-weighted MRI demonstrating resection without (B) and with (C) contrast (arrows). MRI: magnetic resonance imaging

In case 3, an 85-year-old man with metastatic clear cell renal cell carcinoma underwent screening brain MRI prior to starting pazopanib systemic therapy, revealing a solitary 3 cm lesion in the left cerebellar hemisphere with vasogenic edema and mild mass effect (Figure 3A). A left suboccipital craniotomy was performed for tumor resection, given the size of the lesion in the posterior fossa (Figure 3B, 3C). Pathology confirmed metastatic clear cell carcinoma. Postoperative recovery was uneventful. Nine-month brain MRI showed no recurrence. Follow-up imaging at two years showed no evidence of recurrence. He died approximately four years after surgery from heart failure related to progressive systemic disease.

## Discussion

Craniotomy for intracranial metastases in patients over 80 years old is associated with increased risk, primarily due to systemic issues and medical comorbidities, as demonstrated in our series. Surgical intervention may provide benefit in selected patients, particularly those presenting with large symptomatic tumors, well-controlled systemic disease, and limited comorbidities. Our findings align with previous research emphasizing the importance of appropriate patient selection for elderly and very elderly patients to optimize outcomes and minimize risks. One matched-cohort analysis comparing patients over 80 to those under 65 with a range of tumor pathology (both benign and malignant, not separating brain metastases) found no significant differences in 30-day mortality or complication rates after craniotomy when adjusted for factors such as KPS and medical risk [10]. One recent prospective study of 104 patients 65 years and older who underwent craniotomy for brain metastases found favorable morbidity and mortality rates, suggesting that neurosurgical intervention is feasible and safe for this demographic with careful perioperative assessment and management [12]. Further evidence is seen in another series of patients undergoing repeated resections for brain metastases that showed comparable rates of mortality in patients 65 and older versus the general population, with comparable outcomes being attributed to good preoperative functional status in the older group [13].

In this series, neurological tolerance of craniotomy was consistently demonstrated, with favorable postoperative neurological outcomes achieved in all patients. Even in the single case complicated by mild aphasia secondary to a postoperative hematoma, full recovery was observed over time. These findings underscore that advanced age alone should not be considered an absolute contraindication for surgical intervention when neurological function can be preserved. The ability to maintain or improve neurological status postoperatively reflects advancements in surgical technique and perioperative care, supporting the feasibility of craniotomy for intracranial metastases in patients over 80 years old.

Despite these benefits of the local control, long-term survival was limited by systemic disease progression and medical frailty. Radiographic recurrence did not occur, and no deaths were related to intracranial disease progression. Early mortality in our series was attributed to extracranial disease, underscoring the impact of systemic illness on outcomes. Mortality at three months in our group was 2/6 (33%), similar to mortality in three months from another group of very elderly patients undergoing radiosurgery, only 2/8 (25%) [14].

These results highlight that while craniotomy can preserve neurological function and quality of life (such as eventual discharge home) in carefully selected very elderly patients, the procedure carries substantial risk of early mortality related to systemic factors. Thus, surgical candidacy should be determined by multidisciplinary assessment of tumor burden, systemic disease control, and comorbidity profile to optimize benefit and mitigate risk in this vulnerable population.

A key limitation of our study is the small sample size (N=6) and retrospective, single-center design, which limits the generalizability of our findings and makes them susceptible to selection bias. We acknowledge that the patients in this series may represent a highly selected, healthier subset of the very elderly population with brain metastases.

## Conclusions

Historically, craniotomy for tumor resection in the very elderly (≥80 years) was often viewed as high-risk and less favorable due to age-associated comorbidities. Nevertheless, as the elderly population with metastatic brain tumors expands, carefully selected patients may benefit substantially from surgical intervention. Surgery may be reasonable in a patient with a good KPS, a large symptomatic tumor in a non-eloquent area, controlled systemic disease as assessed by CT of the chest/abdomen and pelvis, and acceptable medical risk on formal risk stratification. Our case series highlights that meaningful neurological recovery may be achieved and quality of life maintained postoperatively in some of these patients. However, it remains critical to acknowledge the significant risk of early postoperative mortality driven by systemic disease and medical frailty, as demonstrated by the three patients who died within 2-3 months following surgery. Future prospective multicenter studies should further delineate clear selection criteria for the very elderly with brain metastases and should consider validated quality of life outcome measures. Chronological age alone should not preclude surgical consideration.

## References

[1] Nayak L, Lee EQ, Wen PY (2012). Epidemiology of brain metastases. Curr Oncol Rep.

[2] Ruiz-Garcia H, Marenco-Hillembrand L, Peterson JL (2020). The management of elderly patients with brain metastases from breast cancer. Transl Cancer Res.

[3] Sankey EW, Tsvankin V, Grabowski MM (2019). Operative and peri-operative considerations in the management of brain metastasis. Cancer Med.

[4] Patchell RA, Tibbs PA, Walsh JW (1990). A randomized trial of surgery in the treatment of single metastases to the brain. N Engl J Med.

[5] Martin-Loeches I, Guia MC, Vallecoccia MS (2019). Risk factors for mortality in elderly and very elderly critically ill patients with sepsis: a prospective, observational, multicenter cohort study. Ann Intensive Care.

[6] Grossman R, Mukherjee D, Chang DC, Purtell M, Lim M, Brem H, Quiñones-Hinojosa A (2011). Predictors of inpatient death and complications among postoperative elderly patients with metastatic brain tumors. Ann Surg Oncol.

[7] Seicean A, Seicean S, Schiltz NK, Alan N, Jones PK, Neuhauser D, Weil RJ (2013). Short-term outcomes of craniotomy for malignant brain tumors in the elderly. Cancer.

[8] Lv Z, Zhang W, Zhang Y, Zhong G, Zhang X, Yang Q, Li Y (2022). Metastasis patterns and prognosis of octogenarians with metastatic breast cancer: a large-cohort retrospective study. PLoS One.

[9] Rautalin I, Schwartz C, Niemelä M, Korja M (2021). Mortality of surgically treated 80-year-old or older intracranial meningioma patients in comparison to matched general population. Sci Rep.

[10] Santiago RA, Ali A, Ibrahim B (2024). Safety of craniotomy for brain tumor resection in octogenarians and older patients - a matched - cohort analysis. Int J Neurosci.

[11] Chen L, Shen C, Redmond KJ (2017). Use of stereotactic radiosurgery in elderly and very elderly patients with brain metastases to limit toxicity associated with whole brain radiation therapy. Int J Radiat Oncol Biol Phys.

[12] Lenga P, Scherer M, Kleineidam H, Unterberg A, Krieg SM, Dao Trong P (2025). Neurosurgical management of brain metastases in the elderly: a prospective study on adverse event prevalence and predictors. Neurosurg Rev.

[13] Goldberg M, Heinrich V, Altawalbeh G (2024). The role of repeated surgical resections for recurrent brain metastases in older population. Medicina (Kaunas).

[14] Rades D, Delikanli C, Blanck O, Schild SE, Janssen S (2022). A survival score for very elderly patients with brain metastases assigned to stereotactic radiosurgery or fractionated stereotactic radiotherapy. Anticancer Res.

